# Supplementary material for: Identifying Core Items of the Japanese Version of the Mother-to-Infant Bonding Scale for Diagnosing Postpartum Bonding Disorder
Source: Healthcare (Basel). 2023 Jun 13;11(12):1740. doi: 10.3390/healthcare11121740 (PMC10298576; doi:10.3390/healthcare11121740)
Supplement: Supplementary file 1 [file healthcare-11-01740-s001.zip › healthcare-2340193-supplementary.pdf]

File format: MS Word document (.doc format)

Supplementary Table S1. Factor Loadings of Five MIBS-J Items for Each Factor within Different Models

	MIBS-J item	Time	Parent	Model 1 (1-factor)	Model 2 (2-factor)	
				I	I	II
1	I feel loving toward my child	W1	Fathers (n = 166)	**0.68**	**0.70**	–0.06
			Mothers (n = 282)	**0.68**	**0.73**	–0.10
		W2	Fathers	**0.80**	**0.89**	–0.19
			Mothers	**0.83**	**0.88**	–0.04
		W3	Fathers	**0.70**	**0.72**	–0.08
			Mothers	**0.75**	**0.68**	0.08
2	I feel scared or panicky when I have to do something for my child	W1	Fathers	0.15	–0.03	**1.00**
			Mothers	0.24	–0.02	**1.00**
		W2	Fathers	0.11	–0.09	**0.58**
			Mothers	0.24	–0.15	**0.55**
		W3	Fathers	0.21	0.00	**1.00**
			Mothers	0.20	–0.06	**0.36**
6	I enjoy doing things with my child	W1	Fathers	**0.74**	**0.74**	0.00
			Mothers	**0.60**	**0.53**	0.22
		W2	Fathers	**0.60**	**0.55**	0.11
			Mothers	**0.56**	0.14	**0.63**
		W3	Fathers	**0.62**	**0.60**	0.06
			Mothers	**0.55**	0.04	**0.80**

8	I feel protective toward my child	W1	Fathers	**0.58**	**0.60**	–0.10
			Mothers	**0.64**	**0.66**	–0.05
		W2	Fathers	**0.74**	**0.75**	–0.09
			Mothers	**0.66**	**0.76**	–0.14
		W3	Fathers	**0.78**	**0.80**	–0.06
			Mothers	**0.65**	**0.79**	–0.14
10	I feel close to my child	W1	Fathers	**0.69**	**0.66**	0.17
			Mothers	**0.68**	**0.67**	0.02
		W2	Fathers	**0.66**	**0.57**	**0.36**
			Mothers	**0.69**	**0.50**	0.26
		W3	Fathers	**0.76**	**0.74**	0.09
			Mothers	**0.64**	**0.59**	0.07

MIBS-J = mother-to-infant bonding scale; W1 = 5 days after childbirth; W2 = 1 month after childbirth; W3 = 4 months after childbirth; n = 166 for fathers and 282 for mothers; Factor loadings >0.30 are indicated in boldface; the upper figure in each cell represents factor loading (or total variance explained) among fathers, whereas the lower figure in each cell represents factor loading (or total variance explained) among mothers. Item scores after log transformation were entered into an exploratory factor analysis. Each score ranges from 1 (not at all) to 4 (very much). No items were reversed.

File format: MS Word document (.doc format)

Supplementary Table S2. Measurement and Structural Invariance of Four MIBS-J Items between Fathers and Mothers

Models		χ^2	*df*	χ^2/df	$\Delta\chi^2$ (*df*)	CFI	ΔCFI	RMSEA	ΔRMSEA	Judgement
W1 fathers vs.	Configural	16.179	4	4.045	Ref	0.983	Ref	0.058	Ref	Accept
mothers	Metric	21.976	7	3.139	5.797(3) NS	0.979	0.004	0.049	Δ0.009	Accept
	Scalar	48.216	11	4.383	26.24(4) ***	0.948	0.031	0.062	0.013	Reject
W2 fathers vs.	Configural	17.203	4	4.301	Ref	0.986	Ref	0.061	Ref	Accept
mothers	Metric	18.906	7	2.701	1.703(3) NS	0.988	+0.002	0.044	Δ0.017	Accept
	Scalar	56.213	11	5.110	37.307(4) ***	0.953	0.035	0.068	0.024	Reject
W3 fathers vs.	Configural	8.820	4	2.205	Ref	0.994	Ref	0.037	Ref	Accept
mothers	Metric	28.868	7	4.124	20.048(3) ***	0.974	0.020	0.059	0.22	Reject
	Metric (partial invariance) item 1	23.324	6	3.887	14.504(2) ***	0.980	0.014	0.057	0.020	Reject
	Metric (partial invariance) item 6	28.107	6	4.685	19.287(2) ***	0.974	0.020	0.064	0.027	Reject
	Metric (partial invariance) item 10	10.114	6	1.686	1.294(2) NS	0.995	+0.001	0.028	Δ0.009	Accept
	Scalar	73.648	15	6.695	63.534(9) ***	0.926	0.069	0.103	0.075	Reject

MIBS-J = Japanese modification of the mother-to-infant bonding scale; *df* = degree of freedom; **p* <.05, ***p* <.01, ****p* <.001; Ref = reference; CFI = comparative fit index; RMSEA = root mean square error of approximation; NS = not significant; W1 = 5 days after childbirth; W2 = 1 month after childbirth; W3 = 4 months after childbirth; n = 350 for fathers and 543 for mothers
